# Deletion of transcription factor binding motifs using the CRISPR/spCas9 system in the β-globin LCR

**DOI:** 10.1042/BSR20170976

**Published:** 2017-08-02

**Authors:** Yea Woon Kim, AeRi Kim

**Affiliations:** Department of Molecular Biology, College of Natural Sciences, Pusan National University, Busan 46241, Republic of Korea

**Keywords:** binding motifs, CRISPR/spCas9, transcription factors

## Abstract

Transcription factors play roles in gene transcription through direct binding to their motifs in genome, and inhibiting this binding provides an effective strategy for studying their roles. Here, we applied the CRISPR (clustered, regularly interspaced, short palindromic repeat)/spCas9 (CRISPR-associated protein 9) system to mutate the binding motifs of transcription factors. Binding motifs for erythroid-specific transcription factors were mutated in the locus control region (LCR) hypersensitive sites (HSs) of the human β-globin locus. Guide RNAs targetting binding motifs were cloned into lentiviral CRISPR vector containing the *spCas9* gene, and transduced into MEL/ch11 cells carrying human chromosome 11. DNA mutations in clonal cells were initially screened by quantitative PCR (qPCR) in genomic DNA and then clarified by sequencing. Mutations in binding motifs reduced occupancy by transcription factors in a chromatin environment. Characterization of mutations revealed that the CRISPR/spCas9 system mainly induced deletions in short regions of <20 bp and preferentially deleted nucleotides around the fifth nucleotide upstream of Protospacer adjacent motifs (PAMs). These results indicate that the CRISPR/Cas9 system is suitable for mutating the binding motifs of transcription factors, and, consequently, would contribute to elucidate the direct roles of transcription factors.

## Introduction

Transcription factors regulate the transcription of target genes by binding to motifs present in regulatory regions of the genome. The roles of transcription factors have been elucidated by inhibiting their expressions in a cellular environment [1,2] or by mutating their binding motifs in extrachromosomal loci [3–7]. Such expressional inhibition can demonstrate their direct roles, but cannot exclude indirect effects because transcription factors often affect the expressions of other transcription factors. Thus, mutation at binding motifs can better reveal the direct roles of transcription factors. However such mutations have not been commonly tried in a genome context because of technical difficulties in genome editing. Furthermore, binding motifs for different transcription factors are closely located in regulatory regions such as enhancers or promoters.

Recently, an effective genome editing tool was developed from bacterial adaptive immune system type II, clustered, regularly interspaced, short palindromic repeats (CRISPR) [8,9]. In the bacterial CRISPR system, CRISPR RNA (crRNA) hybridizes with *trans*-activating crRNA (tracrRNA) [10]. The crRNA–tracrRNA duplex directs CRISPR-associated protein 9 (Cas9) endonuclease into target sequences by base-pairing crRNA with DNA [11]. DNA cleavage by Cas9 requires a Protospacer adjacent motif (PAM) next to target sequences [8,11,12]. In the CRISPR/Cas9 system for genome editing, the crRNA–tracrRNA duplex has been simplified to a single guide RNA (sgRNA) [8,13], which usually contains 20 nts for base pairing with target sequences. sgRNA-directed Cas9 has been shown to generate double-stranded breaks in the target sequences [8,11]. These breaks are repaired in a non-homologous end joining manner, resulting in mutations in target sequences [13–15]. The CRISPR/Cas9 system has been used for knocking out genes by disrupting coding regions in various cell lines and organisms [16–22].

spCas9 derived from *Streptococcus pyogenes* uses 5′-NGG-3′ sequences as PAMs [8,23]. These sequences occur every eight bases in the human genome [13], which suggests that almost all the genome sequences can be cleaved by the spCas9. Genome targetting using spCas9 gives rise to the deletion of <10 bp in most cases and the insertion or substitution of short sequences in low frequency [16,24]. Here, we applied the CRISPR/spCas9 system to mutate the binding motifs of transcription factors in a genome context, because these motifs are typically ∼10 bp [25]. The locus control region (LCR) hypersensitive sites (HSs) of the human β-globin locus were used as model regulatory regions, which contains binding motifs for tissue-specific transcription factors such as GATA-1, TAL1, and KLF1 [26]. To clearly analyze changes by genome editing, we employed mouse MEL/ch11 cells that contain a human chromosome 11 where the β-globin locus is present [27]. We found that the CRISPR/spCas9 system can effectively mutate the binding motifs of transcription factors in a genome context and believe that it will contribute to studies on the direct roles of transcription factors.

## Materials and methods

### Cloning guide sequences of sgRNA into CRISPR vectors

Guide sequences of sgRNA were cloned into lentiCRISPRv2 vector (Addgene #52961) [18] as suggested by the manufacturer, but with modifications in the digestion and dephosphorylation steps. To use the BsmBI site for cloning in lentiCRISPRv2 vector, ‘CACCG’ were added to the 5′ end of oligonucleotides for guide sequences, and ‘AAAC’ and ‘C’ were added to the 5′ and 3′ ends of oligonucleotides for complementary sequences, respectively. A pair of oligonucleotides (10 nM) was phosphorylated with T4 polynucleotide kinase (NEB M0201S) at 37°C for 1 h in 10 μl of reaction volume, annealed by heating to 95°C for 5 min and cooling to 25°C at 0.5°C/min, and then diluted to 1:1000 in sterile water. One microgram of lentiCRISPRv2 vector was digested and dephosphorylated with FastDigest BsmBI (Thermo Scientific FD0454) and FastAP (Thermo Scientific EF0651) at 37°C for 30 min in 20 μl of reaction volume. Fifty nanograms of digested vector and 1 μl of diluted oligonucleotide duplex were ligated with Quick T4 DNA ligase (NEB M2200S) at 25°C for 20 min in 11 μl of reaction volume. Ligated plasmid vectors were introduced into competent Stbl3 bacteria. For cloning into pLH-spsgRNA2 vector (Addgene #64114) [28], oligonucleotides were synthesized by adding ‘ACCG’ to the 5′ end of target sequences and ‘AAAC’ to 5′ end of complementary sequences. This pair of oligonucleotides was phosphorylated and annealed as described above, and then cloned into BbsI (NEB R0539S) site in pLH-spsgRNA2 vector. Ligated plasmid vectors were introduced into competent Stbl3 bacteria. All plasmid vectors were prepared using Plasmid Mini Kits (Qiagen). The EGFP sequences (5′-GGGCGAGGAGCTGTTCACCG-3′) were used as control guide sequences [18].

### Cell culture and lentiviral transduction

MEL/ch11 and 293FT cells were cultured in DMEM medium (Gibco) supplemented with 10% FBS (Gibco) and 1% penicillin-streptomycin (Gibco). To produce lentivirus, 0.5 μg lentiviral vector and 1.5 μg packaging mix (pLP1, pLP2, and pLP/VSVG) were diluted in 125 μl Opti-MEM (Gibco), and 6 μl Lipofectamine 2000 (Invitrogen) in another 125 μl Opti-MEM. After incubation for 5 min at room temperature, they were mixed and incubated for an additional 20 min. The DNA-Lipofectamine 2000 mixture was added to 1 × 10^6^ 293FT cells in six-well plate and medium containing the mixture was changed with complete medium the next day. At 72 h after transfection, lentiviral supernatants were harvested and filtered through a 0.45-μm membrane. MEL/ch11 cells were infected with lentiviral supernatants in the presence of 6 μg/ml polybrene (Sigma) and medium was replaced with fresh one the next day. Cells were selected using 2 μg/ml puromycin (Sigma) for lentiCRISPRv2 vector or 500 μg/ml hygromycin (Invitrogen) for pLH-spsgRNA2 vector at 72 h after transduction.

### Screening of mutant cell clones

Clonal cells were grown in 96-well plates after antibiotics selection, and then transferred to 24-well plates when the cell numbers reached 1 × 10^5^ per well. To screen clones with mutations in sequences targetted by sgRNA, genomic DNA was isolated using a QIAamp DNA Mini Kit (Qiagen) from 1 × 10^6^ cells, and amplified by quantitative PCR (qPCR) using the following parameters: 20 ng genomic DNA, 4.5 μM forward primer, 4.5 μM reverse primer, and 1× Power SYBR Green PCR master mix (Applied Biosystems) in 10 μl of reaction volume using the 7300 real-time PCR system (Applied Biosystems). Dissociation curves of qPCR were analyzed using qPCR program and PCR products were visualized on agarose gels. Mutations were clarified by DNA sequencing analysis. The sequences of primers used for PCR are presented in Supplementary Table S1.

### ChIP

Clonal MEL/ch11 cells were cultured at 1.5 × 10^5^ cells per ml in 5 mM HMBA (Sigma) for 72 h to activate the β-globin locus. To cross-link protein to genomic DNA, 1 × 10^7^ cells were incubated in culture medium containing 1% formaldehyde (Sigma) at 25°C for 10 min with shaking, and then glycine (Invitrogen) was added to a final concentration of 0.125 M. After PBS washing, cross-linked cells were incubated in cell lysis buffer (10 mM Tris, 10 mM NaCl, 0.2% NP-40, pH 8.0) at 4°C for 10 min and centrifuged at 600 ***g*** for 5 min to isolate nuclei. The nuclei were treated with 100 U of MNase (Worthington) at 37°C for 15 min, incubated in nuclei lysis buffer (50 mM Tris, 10 mM EDTA, 1% SDS) at 4°C for 10 min and then sonicated to digest chromatin to mainly mononucleosomes. After preclearing with protein G agarose beads (Millipore), chromatin was made to react with antibodies at 4°C for 3 h, and protein–DNA complexes were collected using protein G agarose beads. DNA was purified by phenol extraction and ethanol precipitation and then dissolved in 150 μl of Tris/EDTA buffer. DNA obtained by ChIP was analyzed by qPCR using TaqMan chemistry and the following parameters: 2 μl DNA, 2 μM TaqMan probe, 4.5 μM primers, and 1× TaqMan universal master mix II (Applied Biosystems) in 10 μl of reaction volume using the 7300 real-time PCR system (Applied Biosystems). Antibodies used for ChIP were normal goat IgG (sc-2028), GATA-1 (sc-1233), and TAL1 (sc-12984) from Santa Cruz Biotechnology, and KLF1 (ab2483) from Abcam. The sequences of probes and primers used for ChIP assay are presented in Supplementary Table S2.

## Results and discussion

### Application of the CRISPR/spCas9 system to the mutation of the binding motifs of transcription factors in the human β-globin LCR HSs

**Figure 1:**
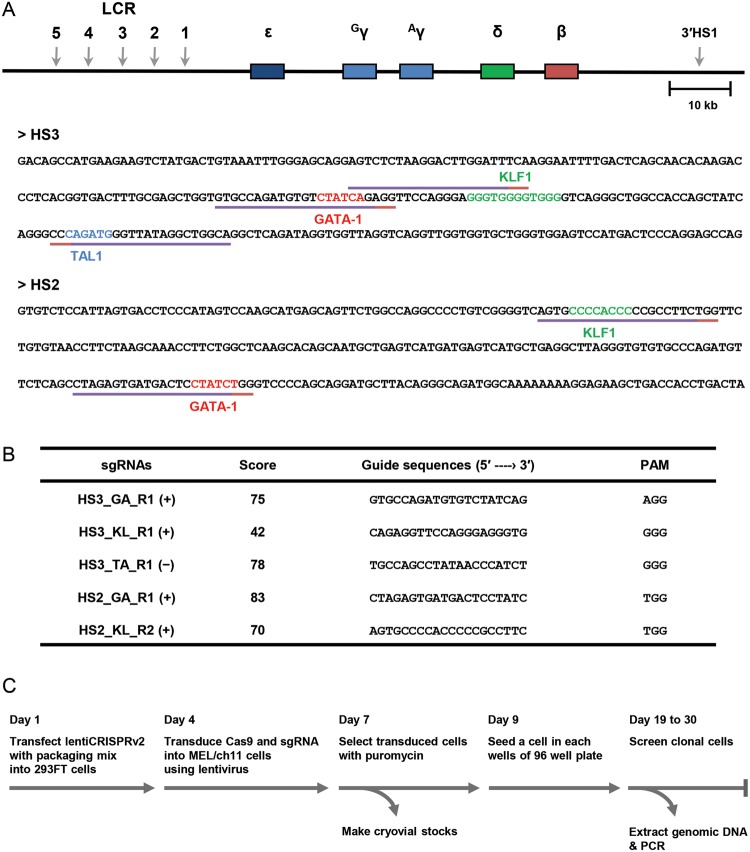
The CRISPR/spCas9 strategy for mutating binding motifs for transcription factors (**A**) The human β-globin locus is presented. Vertical gray arrows indicate DNase I HSs in the LCR and 3′-HS1. The five globin genes, *ε*, *^G^γ*,*^A^γ*, *δ*, and *β* are represented by rectangle in sequence. DNA sequences are for the LCR HS3 and HS2, and the binding motifs of transcription factors are marked by colored bases; GATA-1 motif: red, KLF1 motif: green, and TAL1 motif: blue. Target sequences for sgRNA are indicated by purple lines with PAMs in red lines. (**B**) Guide sequences of sgRNA are listed with names, scores, and PAM sequences, where (+) and (–) mean guide sequences are positive or negative strand DNA, respectively. (**C**) Experimental procedure for mutating binding motifs for transcription factors in MEL/ch11 cells is illustrated and described in ‘Materials and methods’ section.

The human β-globin LCR HSs, which act as enhancers for the β-like globin genes, contain binding motifs for erythroid-specific transcription factors, GATA-1, TAL1, and KLF1 [26]. To mutate these motifs in the LCR HS2 and HS3, we obtained candidate guide sequences for sgRNA in the CRISPR/spCas9 system using an online tool (http://crispr.mit.edu) and chose that their putative cutting sites are located in binding motifs (Figure 1A,B). This tool provides scores for guide sequences that are inversely related to the possibility of binding to off-target DNA. The scores of the chosen guide sequences exceeded 70, except for sequences targetting the KLF1 motif in LCR HS3 (HS3_KL_R1) (Figure 1B). Next, we cloned the guide sequences into lentiCRISPRv2 vector and transduced them into MEL/ch11 cells using lentiviral system (Figure 1C), because suspension cells, like MEL cells, are inefficiently transfected using reagents such as Lipofectamine. Transduced cells were selected using puromycin, diluted, and grown in 96-well plates to obtain clonal cells.

### Screening of mutations generated by the CRISPR/spCas9 system

**Figure 2:**
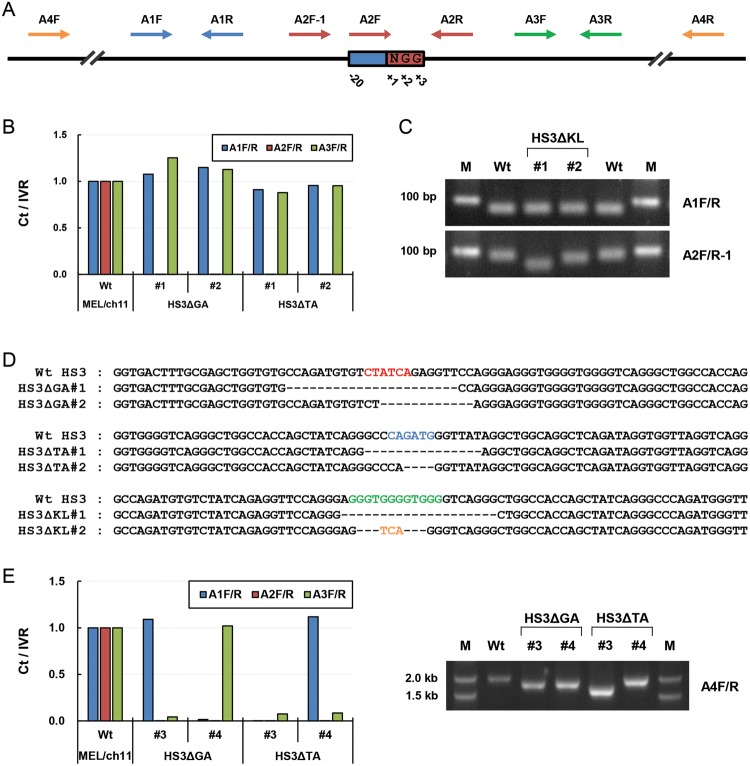
Screening of mutations generated by the CRISPR/spCas9 system (**A**) The locations of primer sets for PCR are presented on DNA with respect to the target sequences of sgRNA. The target sequences are 20 nts in length, and ‘NGG’ nucleotides acting as PAMs are numbered as ‘+1’, ‘+2’, and ‘+3’. One primer in A2 amplicon (red arrows) locates on the target sequences, and A2R-1 primer is on next to the target sequences. A1 (blue arrows) and A3 primers (green arrows) locate in both flanking regions of the target sequences. A4F/R (yellow arrows) primers amplify large region including A1, A2, and A3 amplicons. (**B**) Genomic DNA from clones targetted by the CRISPR/spCas9 system, was amplified by qPCR using the three primer sets, A1, A2, and A3. PCR product amounts were compared using IVR amplicon for intervening region between the LCR and *ε-globin* gene as an internal control and then normalized compared with amounts in wild-type (Wt) MEL/ch11 cells. (**C**) In HS3ΔKL clones, PCR products amplified by A1F/R and A2F/R-1 primers were visualized on an agarose gel, M is the DNA marker. (**D**) DNA sequences in the LCR HS3 are presented for Wt cells and clones with GATA-1, TAL1, or KLF1 motif deletions. The binding motifs of transcription factors are marked by colored bases as described in Figure 1. Black dashes represent deleted nucleotides and yellow bases are inserted nucleotides. (**E**) Genomic DNA of clones was amplified and quantitated as described above. PCR products amplified by A4F/R primers were visualized in an agarose gel.

It is important to screen cell clones that are desirably mutated in target sequences by the CRISPR/spCas9 system. For initial screening, genomic DNA was purified and analyzed by PCR. Primer sets for PCR were designed for guide sequences (A2F/R) and both side regions (A1F/R, A3F/R) (Figure 2A). Figure 2B shows the results of qPCR for wild-type MEL/ch11 cells and clones in which the LCR HS3 GATA-1 motif (HS3ΔGA) or the TAL1 motif (HS3ΔTA) was targetted by guided spCas9. Genomic DNA was similarly amplified using A1 or A3 primer sets in wild-type cells and HS3ΔGA and HS3ΔTA clones. However, A2 primer set did not amplify DNA in HS3ΔGA and HS3ΔTA clones, indicating that mutations occurred in regions annealed by A2 primers (Figure 2B and Supplementary Figure S1A).

To screen clones in which the LCR HS3 KLF1 motif was targetted, A2F-1 primer was designed in the outside region of target sequences because of high GC content in target sequences. Deletions in target sequences were detected by comparing sizes of PCR products (Figure 2C) and by comparing dissociation curves of qPCR (Supplementary Figure S1B). The reduction in PCR product sizes resulted in changes in dissociation curves. Finally mutated regions were identified by DNA sequencing using A4F/R primers. Sequencing results showed that 22 and 12 bp, which include a GATA-1 binding motif, were deleted in HS3ΔGA #1 and #2 clones, respectively (Figure 2D). Mutations of the TAL1 and KLF1 motifs were sequenced in HS3ΔTA and HS3ΔKL clones. These results show that binding motifs for transcription factors can be mutated by sgRNAs of the CRISPR/spCas9 system in a genome context.

On the other hand, we found some clones in which genomic DNA was not amplified by the A1 or A3 primer set in addition to A2 primer set (Figure 2E). It indicates that large regions, including target sequences, were deleted by the attack of guided-spCas9. To determine the approximate lengths of deleted regions, we performed PCR using A4F/R primers, which cover large regions including A1, A2, and A3 amplicons, and ran PCR products in 0.8% agarose gel. It was found that deleted regions had lengths up to 400 bp. Using the same strategy, clones with mutations in the GATA-1 or KLF1 motifs of LCR HS2 were screened and sequenced (Supplementary Figure S2).

The mutations of target sequences can be analyzed using various methods. Mismatch cleavage assay is most commonly used to analyze genome edited by CRISPR/Cas9 [29]. This assay uses heteroduplex DNA that is formed by denaturing and annealing PCR products and digests it using Surveyor nuclease or T7E1 nuclease [29]. However, this assay cannot be used for haploid cells or single chromosomes, such as sex chromosomes, and does not provide information about mutated regions. In contrast with the mismatch cleavage assay, analysis using qPCR can provide information about the locations and approximate lengths of deletions. In addition, many clones can be screened and analyzed in one time by qPCR. Even deletion and/or insertion in a couple of base pair can be missed out in genome screening using qPCR and agarose gel running, this qPCR strategy still provides a simple and efficient means for screening mutant clones for transcription factor binding motifs.

### Deletion of binding motifs for transcription factors inhibited their binding in a chromatin environment

**Figure 3:**
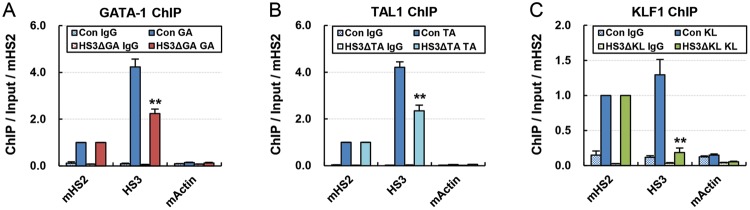
Occupancy of transcription factors in binding motif mutant clones Control cells and clones with GATA-1 (**A**), TAL1 (**B**), or KLF1 (**C**) motif deletion were subjected to ChIP using antibodies specific for GATA-1, TAL1, or KLF1. Amounts of immunoprecipitated DNA were compared with input DNA and then normalized compared with amounts of immunoprecipitated DNA in the mouse β-globin LCR HS2. The mouse *Actin* gene served as an internal negative control and normal goat IgG (IgG) as an experimental negative control. Results are the means ± S.E.M. of two independent experiments; **P<0.01.

To determine whether the deletion of binding motifs results in the loss of transcription factor binding, we carried out ChIP assays on three mutant clones, that are HS3ΔGA#2, HS3ΔTA#1, and HS3ΔKL#2. The HS3ΔGA#2 and HS3ΔTA#1 clones showed decrease in approximately 50% in the occupancies of GATA-1 and TAL1 in the LCR HS3, respectively, as compared with control cells (Figure 3A,B). KLF1 occupancy was more strongly decreased in the HS3ΔKL#2 clone (Figure 3C). The incomplete loss of occupancy for GATA-1 and TAL1 may have been due to other binding motifs near target motifs. Indeed, there are two or more GATA-1 and TAL1 motifs within 200 bp in the human β-globin LCR HS3, whereas only one cluster of KLF1 motifs in the LCR HS3, which was deleted in the HS3ΔKL#2 clone. These results indicate that binding motif deletion can inhibit the binding of transcription factor in a chromatin environment.

### Double deletion of GATA-1 binding motifs in the human β-globin LCR HS3

**Figure 4:**
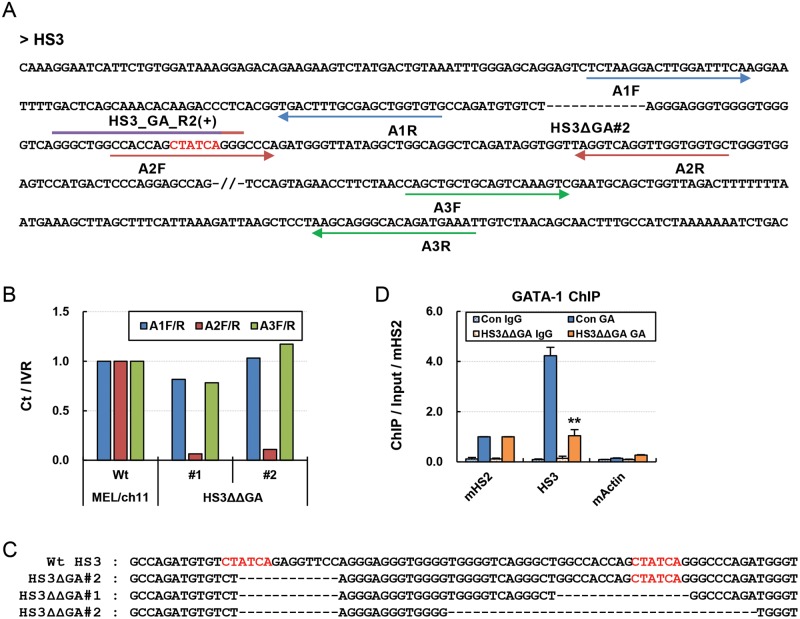
Double deletion of GATA-1-binding motifs in the human β-globin LCR HS3 (**A**) DNA sequences of HS3 in the HS3ΔGA#2 clone are presented with target sequences (purple line) for sgRNA. Another GATA-1 motif is indicated by six red bases. A2F primer (red arrows) is on the GATA-1 motif. (**B**) Genomic DNA was amplified by PCR in the HS3ΔΔGA clones. Amounts of PCR products were measured as described in Figure 2. (**C**) DNA sequences in the LCR HS3 are presented for Wt, HS3ΔGA#2 clone, and HS3ΔΔGA#1 and #2 clones. (**D**) ChIP was performed with antibodies specific for GATA-1 in the HS3ΔΔGA#1. Amounts of immunoprecipitated DNA were determined as described in Figure 3; **P<0.01.

To more strictly inhibit GATA-1 binding in the LCR HS3, we tried to mutate another GATA-1-binding motif located near the first GATA-1 motif. Guide sequences targetting the second GATA-1 binding motif (HS3_GA_R2) were cloned into pLH-spsgRNA2 vector, which has the hygromycin resistance gene as a selectable marker, and then transduced into the HS3ΔGA#2 clone using the procedure described in Figure 1C. After hygromycin selection, clones were analyzed by qPCR using the primer sets shown in Figure 4A. In clone #1 and #2 of HS3ΔΔGA, genomic DNA was not properly amplified by A2 primers (Figure 4B). DNA sequencing revealed that 16 and 37 bp including the second GATA-1 motif were deleted in HS3ΔΔGA #1 and #2, respectively, in addition to the deletion of 12 bp for the first GATA-1 motif (Figure 4C). Finally, we analyzed the occupancy of GATA-1 by ChIP. It was found GATA-1 occupancy was strongly decreased in the LCR HS3 (Figure 4D). These results show that two motifs for the same transcription factor can be sequentially deleted using CRISPR/Cas9 systems with different selection markers, and that this results in greater inhibition of factor binding.

### Characterization of mutations generated by the CRISPR/spCas9 system

**Figure 5:**
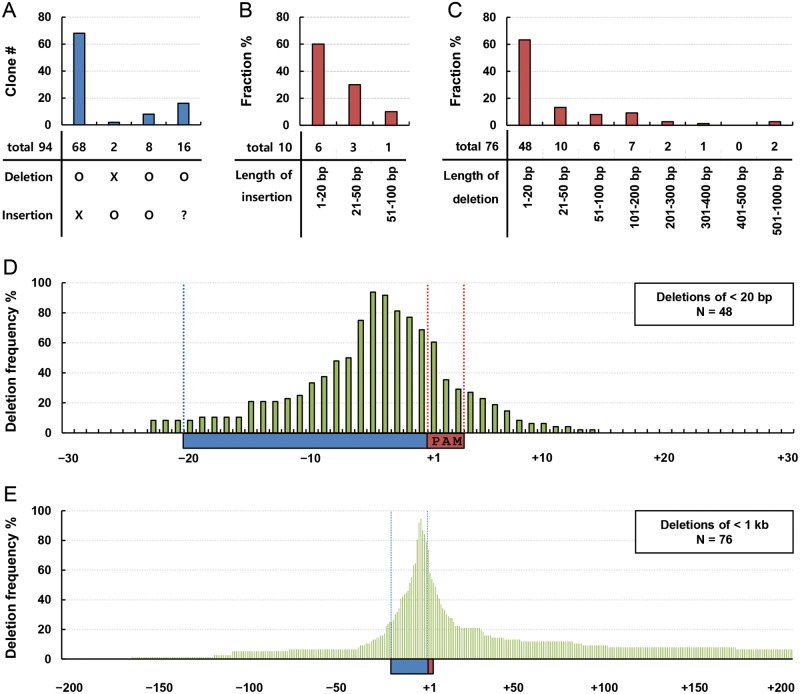
Characterization of mutations generated by the CRISPR/spCas9 system (**A**) Mutations in 94 clones generated using six kinds of sgRNA were sorted by deletion and insertion. Mutations were analyzed by qPCR and/or DNA sequencing. (**B**) Ten clones with an insertion or an insertion and a deletion were sorted depending on the lengths of inserted nucleotides. (**C**) Seventy-six clones with a deletion of <1 kb were sorted depending on the lengths of deleted nucleotides. (**D**) Deletion frequencies were calculated at each nucleotide with respect to target sequences of sgRNA in 48 clones with deletions of <20 bp. The first nucleotide of PAM ‘NGG’ sequences in the red rectangle is indicated by ‘+1’, and nucleotides upstream and downstream of PAMs are indicated by ‘–’ and ‘+’, respectively. (**E**) Using 76 clones with deletions of <1 kb, deletion frequencies were calculated at each nucleotide with respect to target sequences of sgRNA.

The CRISPR/Cas9 technique has evolved over several years and has been utilized to knockout gene expression in many studies [16–22]. To determine whether this genome editing technique using spCas9 is suitable for mutating binding motifs of transcription factors, we analyzed 94 clones that we obtained with six kinds of sgRNA. We could have missed out clones having mutations in a couple of base pairs because of the limited resolution of agarose gels in screening processes. Initially, clones were sorted by the type of mutation (Figure 5A). Sixty-eight clones were found to only exhibit deletion of nucleotides in and near target sequences. Insertion occurred in two clones without deletion and in eight clones with deletion. Sixteen clones had a deletion of a region exceeding 1 kb, as demonstrated by PCR using various pairs of primers. The number of inserted nucleotides was under 20 bp in six out of ten clones (Figure 5B). The lengths of deleted regions were under 20 bp in 48 of 76 clones (Figure 5C). These mutation patterns are similar to those reported previously, but the lengths of deleted regions was longer in our clones as compared with clones in that gene bodies were targetted [14,16]. This may have been due to the open-chromatin structure of the LCR HSs, which make them susceptible to attack by Cas9 endonuclease. In spite of high sensitivity in enhancer regions, our results show that the CRISPR/spCas9 system generates alterations mainly in shorter regions, that is, under 20 bp.

Next, we determined the locations of deleted nucleotides with respect to the guide sequences of sgRNA in 48 clones containing deletions of under 20 bp (Figure 5D). The fourth and fifth nucleotides upstream from PAMs were deleted in the clones over 90%, and several nucleotides near them were deleted in relatively high frequencies. These deletion patterns are consistent with those found in previous studies on plasmids or oligonucleotides, in which a putative cleavage site was identified between the third and fourth nucleotides upstream of PAMs [8,11]. Figure 5E shows the deletion pattern in 76 clones containing deletions of <1 kb. No directional preference was observed to the up or downstream regions from the highly deleted nucleotides between –1 and –6 from PAMs, although the downstream region was deleted further in a small number of clones. This deletion pattern provides guidelines regarding the choice of guide sequences for sgRNA.

In summary, we mutated the binding motifs of transcription factors using the CRISPR/spCas9 system in a genome context, and observed reductions in transcription factor occupancies in a chromatin environment. Our findings imply that the CRISPR/spCas9 system can be used to study the direct roles of transcription factors for target genes without disturbing the expressions of other transcription factors. Furthermore, DNA breaks caused by the CRISPR/spCas9 system resulted mainly in deletions or insertions of less than 20 nts, which indicates that this system is suitable for mutating short *cis*-elements, such as transcription factor binding motifs. Due to this restriction to short regions, other *cis*-elements near target sequences might be maintained during genome editing using the CRISPR/spCas9 system, which would allow study of the individual roles of *cis*-elements. In addition, the deletion of binding motifs for transcription factors using the CRISPR/Cas9 system would be useful for inhibiting the expressions of target genes without side effects, because the mutations of gene coding regions could result in the expression of abnormal polypeptides by nucleotide deletion or frameshift in translation. In recent studies, the *ALAS2* gene transcription was severely inhibited by a GATA-1 motif deletion generated using the CRISPR/Cas9 system [30,31]. Enhancer deletion of the *BCL11A* gene using the CRISPR/Cas9 system suggested the therapeutic possibility for the β-hemoglobinopathies by failing BCL11A expression and resulting in induction of the fetal γ-globin expression [32]. Overall, the CRISPR/Cas9 system is believed to effectively mutate the binding motifs of transcription factors in a genome context, and consequently the mutations can regulate the binding of transcription factors in chromatin and might regulate gene expression.

## Supporting information

**Supplementary Figure 1.** (A)PCR products amplified by A1F/R, A2F/R and A3F/R primers were visualized on agarose gels in HS3ΔGA and HS3ΔTA clones. M is the DNA marker. Dissociation curves generated by qPCR were presented for A1F/R and A2F/R primersets. (B) Dissociation curves of qPCR were presented for A1F/R and A2F/R-1 primer sets inHS3ΔKL clones.

**Supplementary Figure 2.** Screening of mutations generated by the CRISPR/spCas9 system in the β-globin LCR HS2. (A) The locations of primer sets for PCR are presented on DNA with the targets equences of sgRNA and PAMs as described in Figure2. (B) Genomic DNA from clones was amplified by qPCR using the three primer sets, A1, A2 and A3. PCR product amounts were compared using IVR amplicon as an internal control and the nnormalized versus amounts in wild type (Wt) MEL/ch11 cells. (C) DNA sequences in the LCR HS2 are presented for wild type (Wt) cells and clones with GATA-1 or KLF1 motif deletions. The binding motifs of transcription factors are marked by colored bases. Black dashes are deleted nucleotides. (D) Genomic DNA of clones was amplified and quantified as described above. PCR products amplified by A4F/R primers were visualized in an agarose gel.

**Supplementary Table 1:** Sequences of primers for identifying mutations and for sequencing.

**Supplementary Table 2:** Sequences of primers and probes for the ChIP assay.

## Abbreviations

Cas9: CRISPR-associated protein 9
CRISPR: clustered, regularly interspaced, short palindromic repeat
crRNA: CRISPR RNA
DMEM: Dulbecco's modified eagle's medium
EGFP: Enhanced green fluorescent protein
GATA-1: GATA binding protein 1
HMBA: Hexamethylene bisacetamide
HS: hypersensitive site
IVR: Intervening region between the LCR and ε-globin gene
KLF1: Krueppel-like factor 1
LCR: locus control region
MEL/ch11: Murine erythroleukemia cell containing human chromosome 11
MNase: Micrococcal nuclease
PAM: Protospacer adjacent motif
qPCR: quantitative PCR
sgRNA: single guide RNA
TAL1: T-cell acute lympoblastic leukemia 1
tracrRNA: trans-activating crRNA
