# Supplementary material for: Deletion of transcription factor binding motifs using the CRISPR/spCas9 system in the β-globin LCR
Source: Biosci Rep. 2017 Aug 2;37(4):BSR20170976. doi: 10.1042/BSR20170976 (PMC5634328; doi:10.1042/BSR20170976)
Supplement: Supplementary file 1 [file bsr-37-bsr20170976-s1.pdf]

# Supplementary Fig. 1.

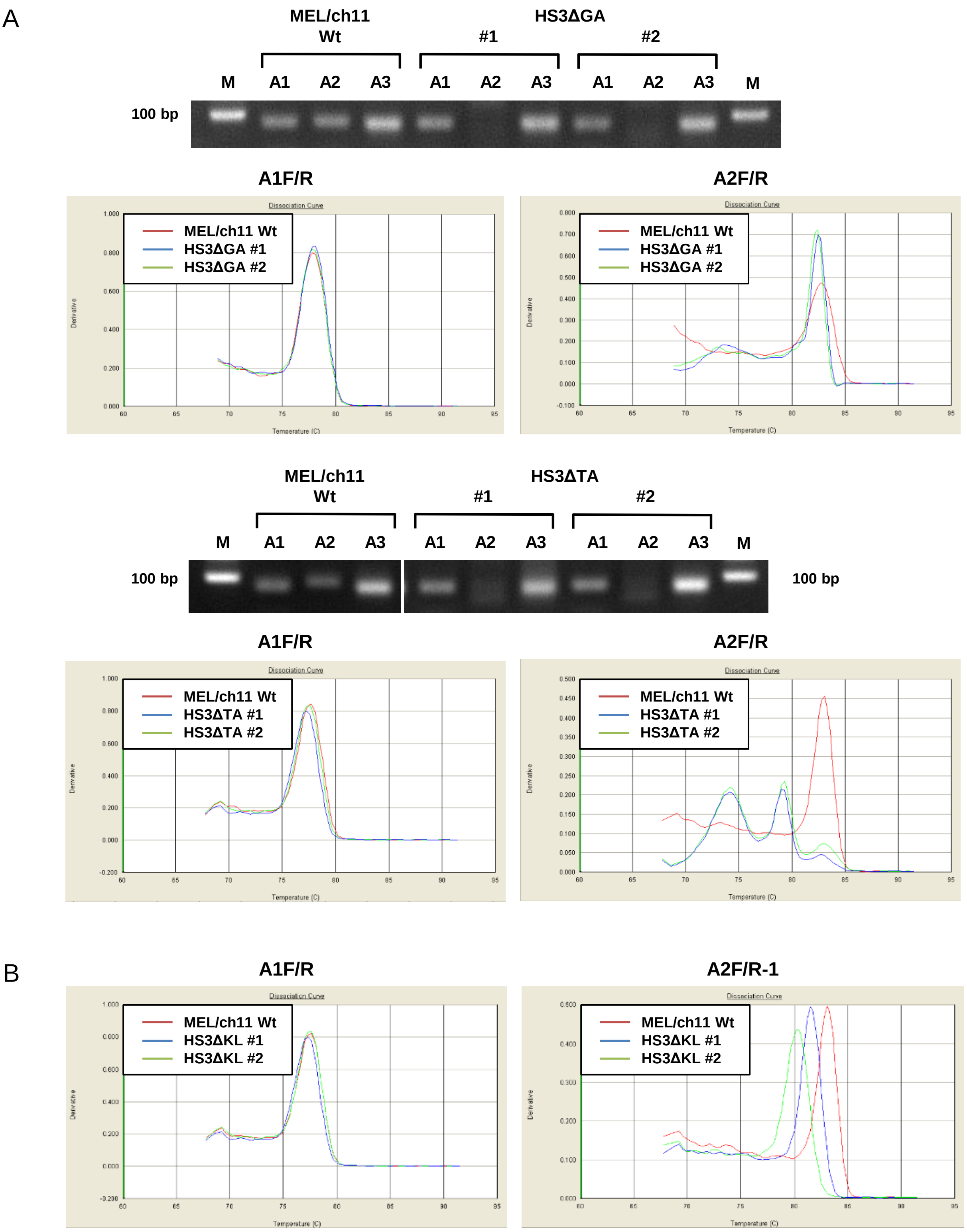


**Supplementary Figure 1.** (A) PCR products amplified by A1F/R, A2F/R and A3F/R primers were visualized on agarose gels in HS3ΔGA and HS3ΔTA clones. M is the DNA marker. Dissociation curves generated by qPCR were presented for A1F/R and A2F/R primer sets. (B) Dissociation curves of qPCR were presented for A1F/R and A2F/R-1 primer sets in HS3ΔKL clones.

Supplementary Fig. 2.

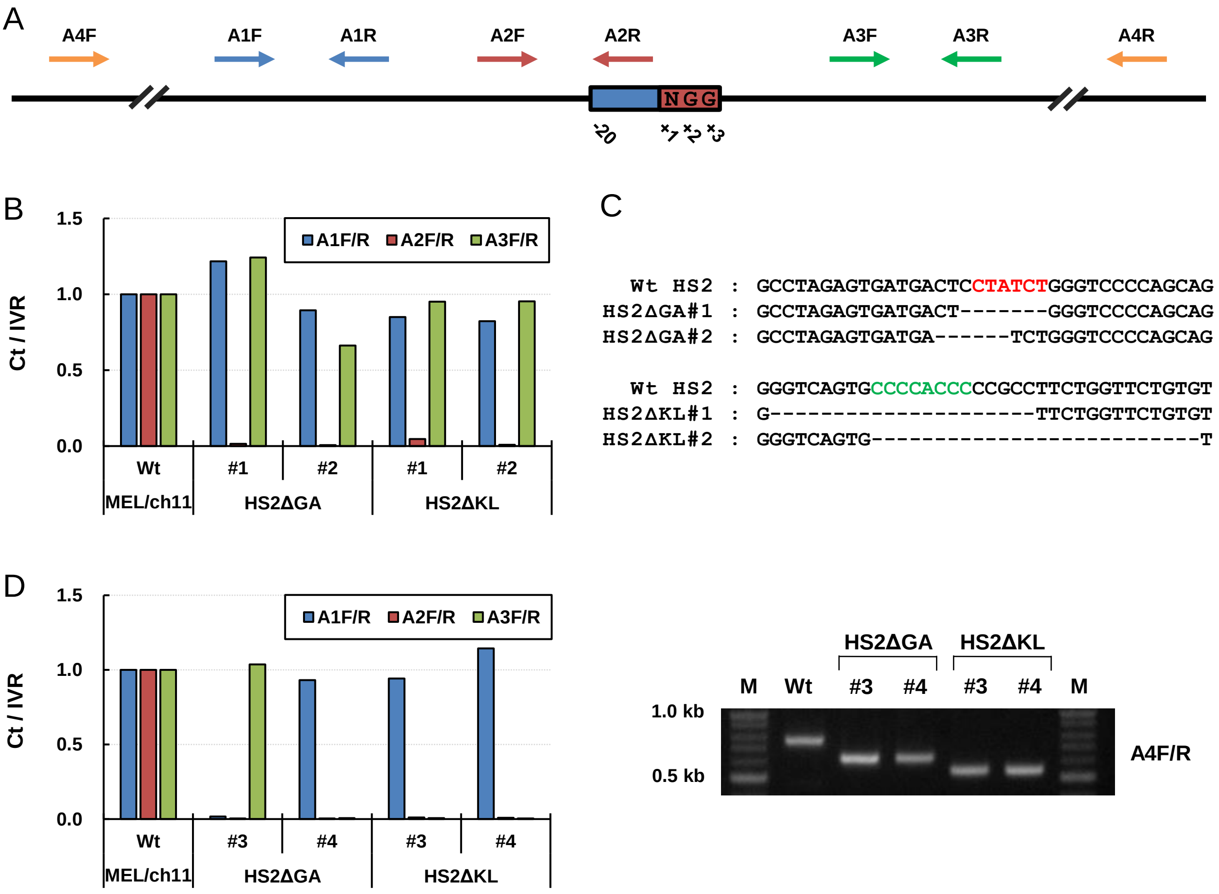


**Supplementary Figure 2.** Screening of mutations generated by the CRISPR/spCas9 system in the β-globin LCR HS2. (A) The locations of primer sets for PCR are presented on DNA with the target sequences of sgRNA and PAMs as described in Figure 2. (B) Genomic DNA from clones was amplified by qPCR using the three primer sets, A1, A2 and A3. PCR product amounts were compared using IVR amplicon as an internal control and then normalized versus amounts in wild type (Wt) MEL/ch11 cells. (C) DNA sequences in the LCR HS2 are presented for wild type (Wt) cells and clones with GATA-1 or KLF1 motif deletions. The binding motifs of transcription factors are marked by colored bases. Black dashes are deleted nucleotides. (D) Genomic DNA of clones was amplified and quantified as described above. PCR products amplified by A4F/R primers were visualized in an agarose gel.

Supplementary Table 1. Sequences of primers for identifying mutations and for sequencing.

| sgRNAs | Amplicons | Primers |
|---|---|---|
| HS3_GA_R1 (+) | A1 | 5' TCT AAG GAC TTG GAT TTC AAG GAA TT 3'<br>5' CAC ACC AGC TCG CAA AGT CA 3' |
| | A2 | 5' CCA GAT GTG TCT ATC AGA GGT TC 3'<br>5' CCA TCT GGG CCC TGA TAG CT 3' |
| | A3 | 5' CCA CCA GCT ATC AGG GCC 3'<br>5' GCA CCA CCA ACC TGA CCT A 3' |
| | A4 | 5' AGG GCT TGG AAA ATC TGT GA 3'<br>5' ATT TCA TCT GTG CCC TGC TT 3' |
| HS3_KL_R1 (+)<br>HS3_TA_R1 (−) | A1 | 5' TCT AAG GAC TTG GAT TTC AAG GAA TT 3'<br>5' CAC ACC AGC TCG CAA AGT CA 3' |
| | A2 | 5' TGT CTA TCA GAG GTT CCA GGG 3'<br>5' CCT GCC AGC CTA TAA CCC AT 3' |
| | A3 | 5' CCA CCA GCT ATC AGG GCC 3'<br>5' GCA CCA CCA ACC TGA CCT A 3' |
| | A4 | 5' AGG GCT TGG AAA ATC TGT GA 3'<br>5' ATT TCA TCT GTG CCC TGC TT 3' |
| HS2_GA_R1 (+) | A1 | 5' CCT CCC ATA GTC CAA GCA TGA 3'<br>5' AGA AGG TTA CAC AGA ACC AGA AG 3' |
| | A2 | 5' GGC TCA AGC ACA GCA ATG C 3'<br>5' CCA GAT AGG AGT CAT CAC TC 3' |
| | A3 | 5' CCT CCC ATA GTC CAA GCA TGA 3'<br>5' AGA AGG TTA CAC AGA ACC AGA AG 3' |
| | A4 | 5' GCT CCC TCT ATC CCT TCC AG 3'<br>5' CAC AGG CCT TTT GCC ACC TA 3' |
| HS2_KL_R2 (+) | A1 | 5' GCT CCC TCT ATC CCT TCC AG 3'<br>5' GCT TGG ACT ATG GGA GGT CA 3' |
| | A2 | 5' CCT CCC ATA GTC CAA GCA TGA 3'<br>5' AGA AGG TTA CAC AGA ACC AGA AG 3' |
| | A3 | 5' GGC TCA AGC ACA GCA ATG C 3'<br>5' CCA GAT AGG AGT CAT CAC TC 3' |
| | A4 | 5' GCT CCC TCT ATC CCT TCC AG 3'<br>5' CAC AGG CCT TTT GCC ACC TA 3' |

Supplementary Table 1. Sequences of primers for identifying mutations and for sequencing. (continued)

| sgRNAs | Amplicons | Primers |
|---|---|---|
| HS3_GA_R2 (+) | A1 | 5' TCT AAG GAC TTG GAT TTC AAG GAA TT 3' |
| | | 5' CAC ACC AGC TCG CAA AGT CA 3' |
| | A2 | 5' CCA CCA GCT ATC AGG GCC 3' |
| | | 5' GCA CCA CCA ACC TGA CCT A 3' |
| | A3 | 5' CAG CTG CTG CAG TCA AAG TC 3' |
| | | 5' ATT TCA TCT GTG CCC TGC TT 3' |
| | A4 | 5' AGG GCT TGG AAA ATC TGT GA 3' |
| | | 5' ATT TCA TCT GTG CCC TGC TT 3' |
| All | IVR | 5' TCC CGA GGT CAG TCA CAC AA 3' |
| | | 5' TGA GGC TCA TTC TCA AGA GTA ATC TC 3' |

Supplementary Table 2. Sequences of primers and probes for the ChIP assay.

| Amplicons | Primers | Taqman probes |
|---|---|---|
| mHS2 | 5' CAG GCG GAG TCA ATT CTC TAC TC 3' | 6FAM CTG TGG GTG TGT TCA |
| | 5' TGC TGT GCT CAA GCC TGA TG 3' | GCC TTG TGA GC TAMRA |
| HS3 | 5' TCT AAG GAC TTG GAT TTC AAG GAA TT 3' | 6FAM TGA CTC AGC AAA CAC |
| | 5' CAC ACC AGC TCG CAA AGT CA 3' | AAG ACC CTC ACG TAMRA |
| mActin | 5' ACC CCA TTG AAC ATG GCA TT 3' | 6FAM TTA CCA ACT GGG ACG |
| | 5' TGT AGA AGG TGT GGT GCC AGA T 3' | ACA TGG AGA A TAMRA |
